# Two‐year follow‐up of infant and maternal outcomes after planned early delivery or expectant management for late preterm pre‐eclampsia (PHOENIX): A randomised controlled trial

**DOI:** 10.1111/1471-0528.17167

**Published:** 2022-05-12

**Authors:** Alice Beardmore‐Gray, Melanie Greenland, Louise Linsell, Edmund Juszczak, Pollyanna Hardy, Anna Placzek, Rachael Hunter, Jenie Sparkes, Marcus Green, Andrew Shennan, Neil Marlow, Lucy C. Chappell, the PHOENIX Study Group

**Affiliations:** ^1^ School of Life Course Sciences King's College London London UK; ^2^ Oxford Vaccine Group University of Oxford Oxford UK; ^3^ National Perinatal Epidemiology Unit Clinical Trials Unit Nuffield Department of Population Health University of Oxford Oxford UK; ^4^ Nottingham Clinical Trials Unit School of Medicine University of Nottingham Nottingham UK; ^5^ Experimental Psychology Unit University of Oxford Oxford UK; ^6^ Research Department of Primary Care and Population Health University College London London UK; ^7^ Action on Pre‐eclampsia Evesham UK; ^8^ Institute for Women's Health University College London London UK

**Keywords:** delivery, infant, neurodevelopment, pre‐eclampsia, preterm

## Abstract

**Objective:**

We evaluated the best time to initiate delivery in late preterm pre‐eclampsia in order to optimise long‐term infant and maternal outcomes.

**Design:**

Parallel‐group, non‐masked, randomised controlled trial.

**Setting:**

Forty‐six maternity units in the UK.

**Population:**

Women with pre‐eclampsia between 34^+0^ and 36^+6^ weeks of gestation, without severe disease, were randomised to planned delivery or expectant management.

**Main outcome measures:**

Infant neurodevelopmental outcome at 2 years of age, using the Parent Report of Children’s Abilities – Revised (PARCA‐R) composite score.

**Results:**

Between 29 September 2014 and 10 December 2018, 901 women were enrolled in the trial, with 450 women allocated to planned delivery and 451 women allocated to expectant management. At the 2‐year follow‐up, the intention‐to‐treat analysis population included 276 women (290 infants) allocated to planned delivery and 251 women (256 infants) allocated to expectant management. The mean composite standardised PARCA‐R scores were 89.5 (SD 18.2) in the planned delivery group and 91.9 (SD 18.4) in the expectant management group, with an adjusted mean difference of −2.4 points (95% CI −5.4 to 0.5 points).

**Conclusions:**

In infants of women with late preterm pre‐eclampsia, the average neurodevelopmental assessment at 2 years lies within the normal range, regardless of whether planned delivery or expectant management was pursued. With the lower than anticipated follow‐up rate there was limited power to demonstrate that these scores did not differ, but the small between‐group difference in PARCA‐R scores is unlikely to be clinically important.

## Abstract 2

**Linked article**: This article is commented on by Susan Walker and Brett Manley, pp. 1664‐1665 in this issue. To view this minicommentary visit https://doi.org/10.1111/1471-0528.17165.

## 1 INTRODUCTION

Pre‐eclampsia complicates between 2% and 3% of pregnancies in high‐income settings,^1^ and is a leading cause of iatrogenic preterm birth.^2^ It is a multisystem disorder characterised by placental and maternal vascular dysfunction and is associated with severe complications for both mother and infant.^3^ Potential adverse consequences include maternal and perinatal death, maternal stroke, renal and hepatic injury and fetal growth restriction. Current management of pre‐eclampsia in most high‐income settings involves the close monitoring of maternal and fetal condition, with delivery recommended at 37 weeks of gestation, or sooner, if there is evidence of severe maternal or fetal compromise.^4^, ^5^ At 37 weeks of gestation, previous trials have shown that the initiation of delivery benefits the woman without any additional perinatal risk.^6^

In women with pre‐eclampsia between 34^+0^ and 36^+6^ weeks of gestation, without severe features of the disease necessitating delivery, there is less evidence to guide the optimal timing of birth.^6^ At this gestation, any maternal or perinatal benefit offered by early delivery must be balanced against the potential short‐ and long‐term impacts of late prematurity to the infant. The PHOENIX trial showed that a policy of routine planned early delivery between 34^+0^ and 36^+6^ weeks of gestation significantly reduces short‐term adverse maternal outcomes.^7^ This was accompanied by an increase in neonatal unit admissions, but the indicators of short‐term neonatal morbidity were similar between groups. Before making firm recommendations to guide clinical practice based upon these findings, it is important to fully evaluate the impact of planned delivery in this group on longer‐term infant outcomes. Planned delivery may improve neurodevelopmental outcomes, as the disease process itself will be stopped, thereby limiting the continuing placental dysfunction associated with fetal growth restriction and other morbidities. However, the consequences of the intervention (planned delivery resulting in an earlier gestational age by 3–5 days, compared with expectant management) could also adversely impact neurodevelopmental outcomes. Thus, there remains a clinical dilemma about the best time to plan delivery, in order to optimise short‐ and long‐term infant outcomes.

The aim of this follow‐up study was to evaluate the primary infant outcomes of the PHOENIX trial at 2 years, comparing neurodevelopmental outcomes for infants of women with late preterm pre‐eclampsia randomised to planned early delivery or to expectant management. Additionally, we evaluated the impact of the intervention on secondary maternal outcomes (health‐related quality of life) and will report on the health economic evaluation separately.

## 2 METHODS

### 2.1 Study design and participants

The PHOENIX trial was a parallel‐group, non‐masked, multicentre randomised controlled trial across 46 maternity units in the UK. The published trial protocol and short‐term co‐primary outcomes described the trial methodology in detail,^7^, ^8^ and therefore a brief summary is provided here. There were no substantial changes to the published study design, methods or outcomes after the start of the trial. The trial was approved by the South Central – Hampshire B Research Ethics Committee (no. 13/SC/0645). We compared planned delivery with expectant management (usual care) in pregnant women presenting with pre‐eclampsia between 34^+0^ and 36^+6^ weeks of gestation, without severe features of the disease (which would necessitate immediate delivery), aged 18 years or older, with a singleton or dichorionic diamniotic twin pregnancy and at least one viable fetus. Women with any other comorbidity or with a previous caesarean section or with any fetal position were eligible. The only exclusion criterion to participation was the clinician’s decision to initiate delivery within the subsequent 48 h. After providing written informed consent, women were randomly assigned to planned delivery or expectant management via a secure web‐based randomisation program provided by MedSciNet. A (non‐deterministic) minimisation algorithm, including study centre, singleton or twin pregnancy, severity of hypertension in the 48 h before enrolment, parity, previous caesarean section and gestational age at randomisation, was used to ensure balance between the groups. The intervention could not be hidden from women, clinicians or data collectors because of the nature of the intervention.

### 2.2 Interventions

Planned early delivery consisted of the initiation of delivery within 48 h of randomisation, to allow for the administration of antenatal corticosteroids if deemed necessary by clinicians. Induction of labour was commenced according to local protocol, with caesarean section undertaken only if an additional obstetric indication was present. Expectant management consisted of usual care, with close monitoring of the maternal and fetal condition, until either 37 completed weeks of pregnancy or the development of severe features necessitating delivery.

### 2.3 Data collection

Baseline and short‐term clinical outcome data were collected up until maternal and infant discharge from hospital and recorded on the web‐based trial database. Long‐term outcomes were assessed at 6 months post‐delivery and again when the infant was 2 years of age. Questionnaires were posted to all woman at these time points (or a link was sent electronically) and participants completed a paper copy or an online version captured by the MedSciNet study database. Health resource use and quality‐of‐life outcomes, including the EQ‐5D‐5L questionnaire, were also collected and are reported separately.

### 2.4 Outcomes

#### 2.4.1 Infant outcomes

The primary long‐term infant outcome was neurodevelopmental assessment at 2 years of age, using the Parent Report of Children’s Abilities – Revised (PARCA‐R) composite score.^9^ Secondary long‐term infant outcomes were the non‐verbal and language PARCA‐R subscale scores. The PARCA‐R is a questionnaire completed by a parent (or caregiver), taking 15 min to complete, that assesses non‐verbal and language development. It is recommended by the National Institute of Health and Clinical Excellence (NICE) as a practical and cost‐effective method of identifying cognitive and language delay at 24 months in children born preterm.^10^ Raw scores from the non‐verbal subscale (range 0–34) and language subscale (0–124) are summed to produce an overall composite score. Non‐verbal PARCA‐R scores were prorated if up to four subscale questions were missing. During the trial the methodology to convert the overall composite score to an age‐ and sex‐adjusted standard score and percentile ranking, relative to the norm, was published,^11^ requiring the questionnaire to have been completed at 2 years corrected age (between 23 months and 16 days and 27 months and 15 days). A standardised score of between 85 and 114 would indicate development in the normal range, with scores between 70 and 84 indicating mild delay, scores between 55 and 69 indicating moderate delay and scores of 54 or less indicating severe delay.

#### 2.4.2 Maternal outcomes

Secondary long‐term maternal outcomes included quality of maternal physical and mental health scored using the validated SF‐12v2 Health Survey, a short‐form generic measure of health status with eight health‐related domains.^12^ Scores from each of the eight health concepts can be used to generate a physical component summary scale score (PCS‐12) and a mental component summary scale score (MCS‐12), both with a mean of 50 and a standard deviation of 10, and with a higher score indicating better health. It has been validated in diverse populations, including women who are postpartum.^13^, ^14^, ^15^, ^16^

For participants who completed the long‐term follow‐up, we have additionally reported the co‐primary short‐term outcome (a composite of maternal morbidity using fullPIERS outcomes and recorded systolic blood pressure of at least 160 mmHg post‐randomisation) and the co‐primary short‐term perinatal outcome (a composite of neonatal deaths within 7 days of delivery and perinatal deaths or neonatal unit admissions).^17^ Outcomes were selected before the development of a core outcome set for pre‐eclampsia, which does not currently include any long‐term outcomes.^18^

### 2.5 Sample size

An initial loss to follow‐up rate of 20% assumed that long‐term outcomes would be available for approximately 690 infants.^8^ This calculation was revised before follow‐up was completed and analysis was undertaken, to take into account the higher than expected loss to follow‐up rate of 40%. Based on this, it was anticipated that long‐term outcomes would be available for approximately 568 infants in total (284 per group, assuming no difference in loss to follow‐up between groups). With a one‐sided significance level of 2.5%, under a non‐inferiority hypothesis, a sample size of 284 in each group achieves 88% power to detect a non‐inferiority margin of difference in the mean PARCA‐R score of no fewer than four points (one‐quarter of a standard deviation). A higher response rate would have enabled narrower confidence intervals and more certainty in our conclusions.

### 2.6 Statistical analysis

Demographics and clinical characteristics at baseline and short‐term infant and maternal outcomes are reported using descriptive statistics. The primary inferences for the 2‐year infant outcomes were based on a non‐inferiority hypothesis testing framework in both the intention‐to‐treat (ITT) and the per‐protocol (PP) analysis populations. The primary inferences for the 6‐month and 2‐year maternal outcomes were based on a superiority hypothesis testing framework in the intention‐to‐treat analysis population. All analyses used the expectant management group as the reference group. There were no interim analyses planned.

#### 2.6.1 Infant outcomes

With the statistical analysis plan based on standardised scores, but with infant questionnaires being sent out at a chronological age of 2 years, a lower proportion than anticipated of PARCA‐R questionnaires were completed during the time window allocated for standardising (at <23.5 and >27.5 months of age, corrected for prematurity). To correct for this, multiple imputation by chained equations was used to impute the PARCA‐R standardised scores for those infants (approximately 74% of responders). Imputation models included the raw PARCA‐R scores, age‐corrected for prematurity, sex, minimisation factors and any auxiliary variables associated with the outcome or the missingness of the outcome. Imputation models were developed separately for each outcome and each population. Pooled estimates were obtained from linear regression models, adjusted for minimisation factors as fixed effects and the correlation between multifetal pregnancies. Centre was not fitted as a random effect as planned, because of model non‐convergence. Pooled adjusted means, adjusted mean differences and 95% confidence intervals are reported. The *p*‐values for the composite score alone are reported, and are for one‐sided 2.5% significance non‐inferiority tests based on a margin of four standardised score points.

#### 2.6.2 Maternal outcomes

Mixed‐effect linear regression models adjusted for minimisation factors were fitted for the maternal outcomes (PCS‐12 and MCS‐12), with centre fitted as a random effect. The adjusted mean values, the adjusted mean differences, the 95% confidence intervals and the corresponding *p*‐values are reported. The means and standard deviations for subdomains are unadjusted.

#### 2.6.3 Subgroup analyses

Pre‐specified subgroup analyses for the 2‐year infant outcomes were performed on the multiply imputed data sets for the composite PARCA‐R score. Pooled estimates were obtained from the same linear regression models used for the primary analysis, containing an interaction term between the subgroup and the study arm. Pooled adjusted means and 95% confidence intervals are reported.

#### 2.6.4 Sensitivity analyses

Sensitivity analyses were performed on the 2‐year infant outcome, excluding infants outside of the time window for standardisation. Mixed‐effect linear regression models were fitted, adjusting for correlation between twins, minimisation factors as fixed effects and centre as a random effect. The adjusted mean values, the adjusted mean differences and the 95% confidence intervals are reported.

### 2.7 Role of the funding source

The study was funded by the UK’s National Institute for Health and Care Research (NIHR) Health Technology Assessment Programme (12/25/03) following external peer review, and with involvement of public representative panel members. The funder of the study had no role in the study design, data collection, analysis, interpretation or writing of the report. The corresponding author had full access to all the data in the study and had final responsibility for the decision to submit for publication. The trial was prospectively registered with the ISRCTN registry (ISRCTN01879376).

### 2.8 Patient and public involvement

We worked with representatives (including those with lived experience of pre‐eclampsia) from Action on Pre‐eclampsia (the patient support group) and Tommy’s (a national baby charity) to ensure that the voices of pregnant women (and their wider families) informed and influenced every stage of the research process. Full details on the methodology and outcomes of this are reported in Table S8 (GRIPP2‐SF checklist) of the supporting information.

## 3 RESULTS

**FIGURE 1:**
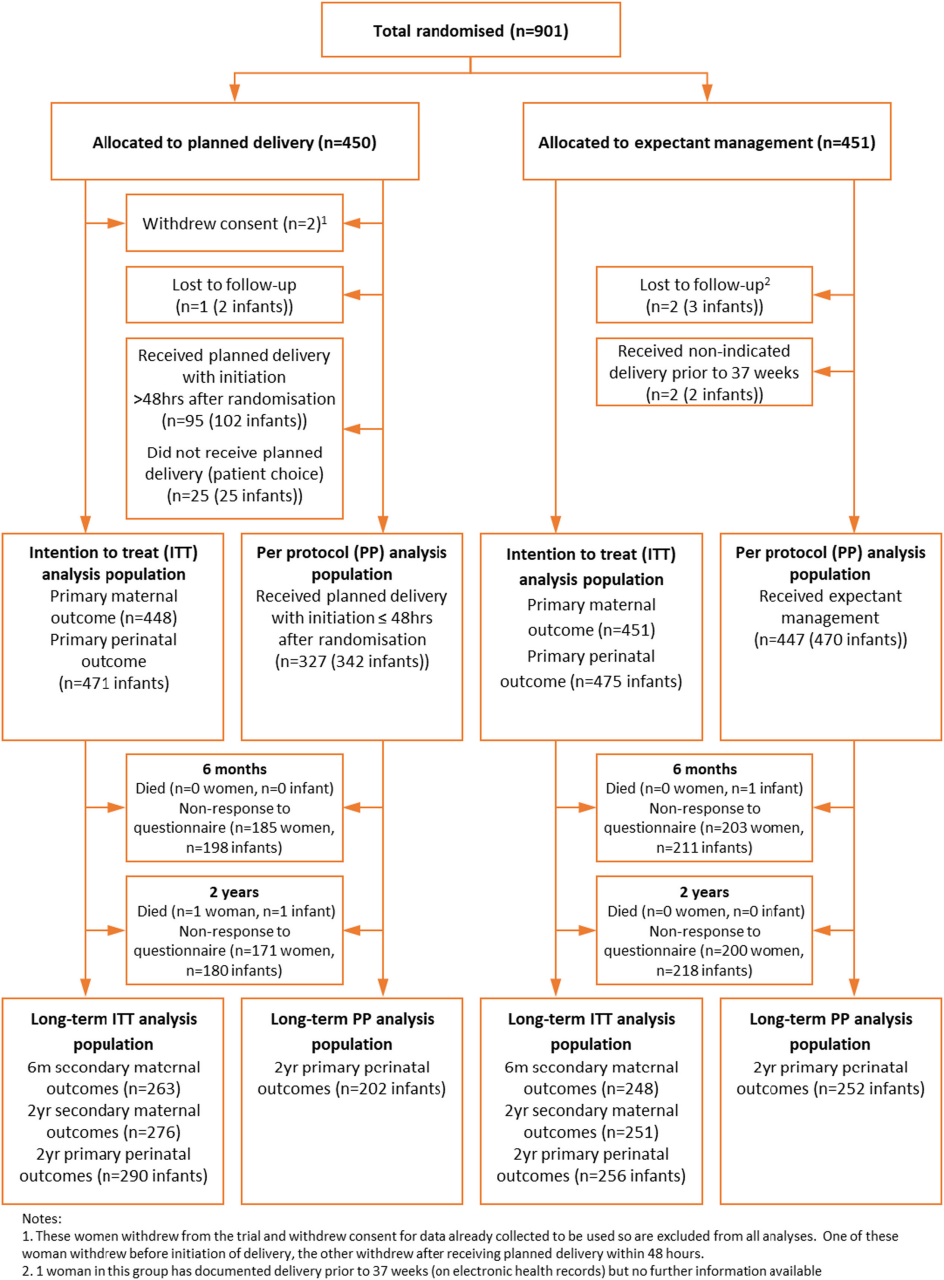
Consolidated Standards of Reporting Trials (CONSORT) flow diagram of participants

Between 29 September 2014 and 10 December 2018, 901 women were enrolled in the trial, with 450 women allocated to planned delivery and 451 women allocated to expectant management (Figure 1). The ITT analysis population for short‐term maternal and perinatal outcomes included 448 women (471 infants) allocated to planned delivery (as two of the allocated women withdrew consent) and 451 women (475 infants) allocated to expectant management. Follow‐up for the 2‐year assessment continued until 31 December 2020. At the 2‐year follow‐up, the long‐term ITT analysis population included 290 infants (62%) and 276 women allocated to planned delivery and 256 infants (54%) and 251 women allocated to expectant management. There were no serious adverse events reported at long‐term follow‐up.

### 3.1 Characteristics of women responding to follow‐up

Baseline maternal and pregnancy characteristics of women responding at 2 years were broadly similar across the two randomised groups (Table 1). The median gestational age at randomisation in both groups was 36 weeks, and the prevalence of suspected growth restriction was similar (19.8% in the planned delivery group and 23.1% in the expectant management group). The study centre at randomisation of the women responding at 2 years is shown in Table S1.

**TABLE 1:** Maternal demographic and pregnancy characteristics

Baseline characteristics	Planned delivery (*n* = 276)	Expectant management (*n* = 251)
Age at randomisation (years), mean (SD)	31.1 (5.7)	31.4 (6.1)
Ethnicity, *n* (%)		
White	200 (72.5)	189 (75.3)
Black	23 (8.3)	21 (8.4)
Asian	42 (15.2)	22 (8.8)
Other	11 (4.0)	19 (7.6)
Deprivation index quintile 5 (most deprived), *n* (%)^a^	79 (30.6)	75 (31.0)
No previous pregnancies ≥24 weeks of gestation), *n* (%)^b^	166 (60.1)	159 (63.3)
Previous caesarean section, *n* (%)^b^	40 (14.5)	43 (17.1)
History of pre‐eclampsia, *n* (%)	50 (18.1)	47 (18.7)
Body mass index at booking (kg/m^2^), mean (SD)	30 (7.6)	29.2 (6.7)
Smoking at booking, *n* (%)	16 (5.8)	16 (6.4)
Systolic BP at booking (mmHg), mean (SD)	119.0 (13.6)	119.5 (13.2)
Diastolic BP at booking (mmHg), mean (SD)	72.8 (10.0)	73.3 (10.21)
Pre‐existing chronic hypersion, *n* (%)	29 (10.5)	33 (13.1)
Pre‐existing chronic renal disease, *n* (%)	3 (1.1)	2 (0.8)
Pre‐pregnancy diabetes, *n* (%)	15 (5.4)	14 (5.6)
Gestational diabetes, *n* (%)	36 (13.0)	21 (8.4)
Aspirin prescribed during pregnancy, *n* (%)	114 (41.3)	101 (40.2)
LMWH prescribed during pregnancy, *n* (%)	69 (25.0)	66 (26.3)
Characteristics at randomisation		
Gestational age at randomisation (weeks), median (IQR)^b^	36 (35–36)	36 (35–36)
Singleton pregnancy, *n* (%)^b^	261 (94.6)	238 (94.8)
Highest systolic BP in previous 48 h (mmHg), mean (SD)	155 (14.8)	155.6 (16.1)
Highest diastolic BP in previous 48 h (mmHg), mean (SD)	95.8 (9.5)	95.8 (11.3)
Highest systolic BP in previous 48 h (mmHg), *n* (%)^b^		
≤149	100 (36.2)	88 (35.1)
150–159	69 (25.0)	65 (25.9)
≥160	107 (38.8)	98 (39.0)
Urinary protein/creatinine ratio ≥30 (mg/mmol), *n* (%)	253 (91.7)	228 (90.8)
Urinary protein/creatinine ratio (mg/mmol), median (IQR)	88 (43–185)	87 (43–197)
Fetal growth restriction ultrasound in previous 2 weeks, *n* (%)	222 (80.4)	212 (84.5)
Suspected fetal growth restriction on ultrasound, *n* (%)	44 (19.8)	49 (23.1)
Inpatient at time of randomisation, *n* (%)	217 (78.6)	210 (83.7)

Abbreviations: BP, blood pressure; LMWH, low molecular weight heparin.

^a^ Deprivation quintiles calculated for participants in England only (not available for participants in Wales).

^b^ Minimisation factors used to ensure balance at randomisation.

In women who completed the 2‐year assessment, a higher proportion of infants in the planned delivery group had been delivered at 34 weeks of gestation (17.2% vs. 11.7%), as expected with the trial intervention (Table S2), and had been admitted to the neonatal unit (40.3% vs. 35.5%), driven by admissions where the primary indication was listed as prematurity. However, a higher proportion of infants in the expectant management group were born small‐for‐gestational age (21.5% vs. 14.1% <10th centile; 5.1% vs. 2.8% <3rd centile), compared with those in the planned delivery group. Maternal mortality and morbidity were lower for responding women allocated to planned delivery, compared with those allocated to expectant management (65.2% vs. 75.5%) (Table S3).

### 3.2 Primary infant outcomes

**FIGURE 2:**
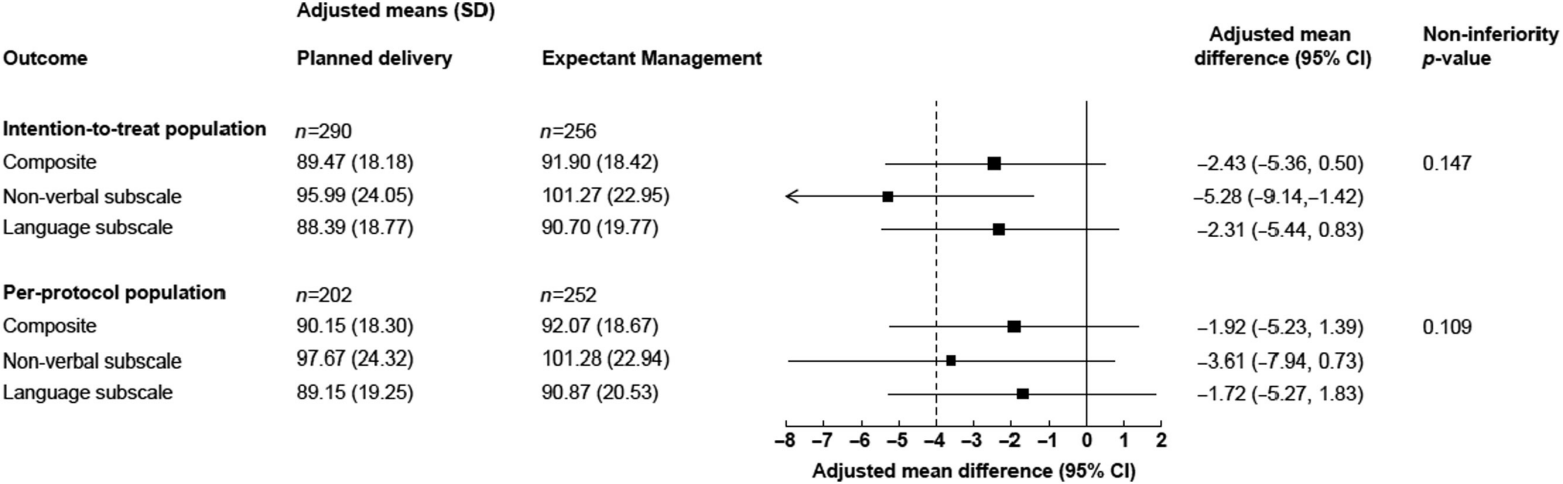
Primary infant long‐term outcome non‐inferiority comparison: imputed standardised Parent Report of Children’s Abilities – Revised (PARCA‐R) at 2 years follow‐up. Standardised scores were imputed for responders who had raw PARCA‐R scores outside of the time window used for standardisation. The *p*‐values are for one‐sided 2.5% significance non‐inferiority tests based on a margin of four standardised score points. The dashed line shows the non‐inferiority margin. The solid line shows the line of no difference. CI, confidence interval; SD, standard deviation

Of the 546 infant questionnaires returned, and using imputed standardised scores for those who had a raw PARCA‐R score outside of the age window for standardisation, the adjusted mean difference comparing planned delivery with expectant management for the composite PARCA‐R score at 2‐years follow‐up was −2.4 (89.5 vs. 91.9, 95% CI −5.4 to 0.5, non‐inferiority *p* = 0.1) in the ITT population (Figure 2). The confidence interval encompassed the four‐point margin and so we could not conclude non‐inferiority. Similar results were seen in the PP population: −1.9 (90.2 vs. 92.1, 95% CI −5.2 to 1.4, non‐inferiority *p* = 0.1) (Figure 2). The adjusted means for both groups and populations were within the range of 85–114 (indicating normal neurodevelopment), as were the adjusted means for the subscale scores (Figure 2).

### 3.3 Maternal outcomes

**FIGURE 3:**
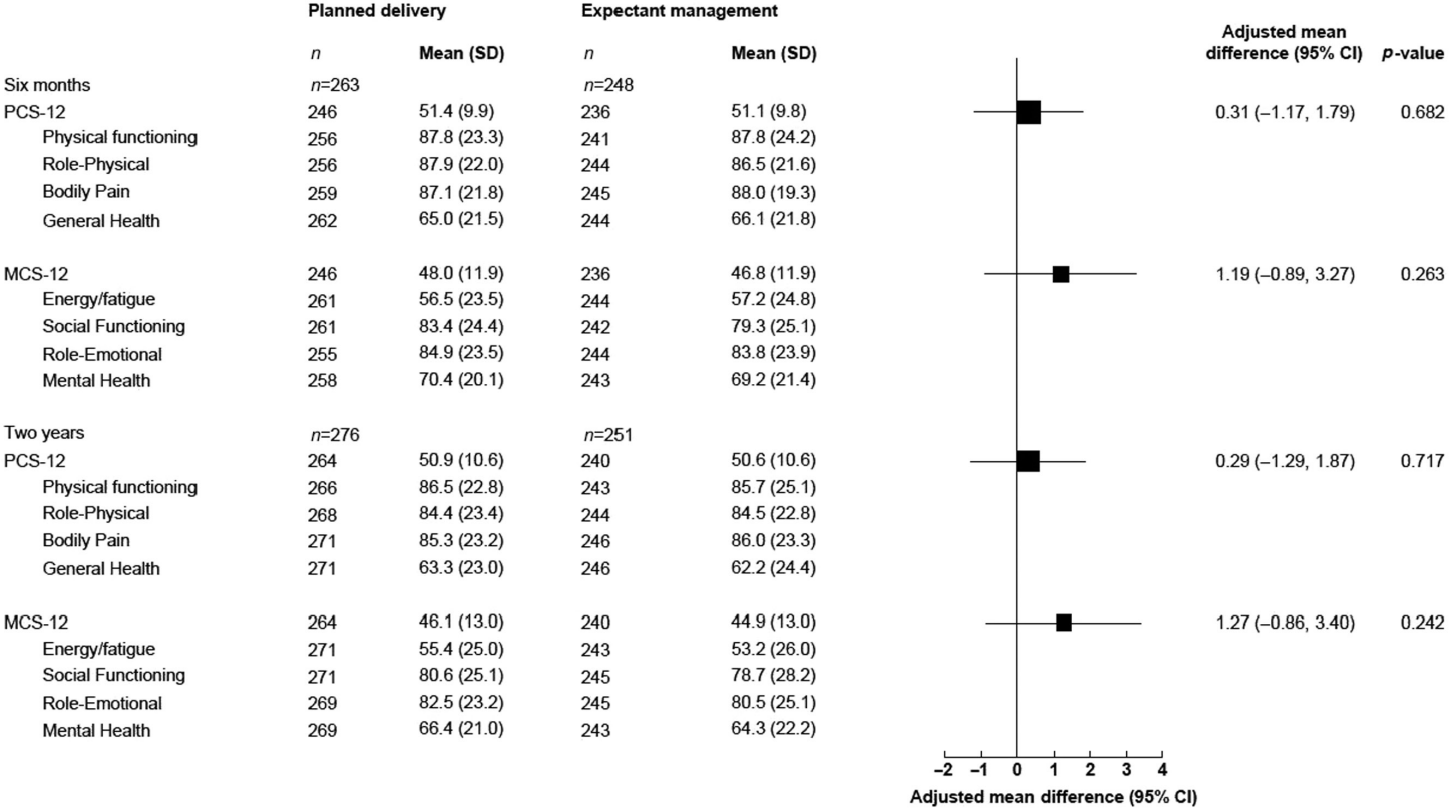
Maternal secondary long‐term outcomes: SF‐12 Health Survey Summary Scale at follow‐up at 6 months and at 2 years. The solid line shows the line of no difference. CI, confidence interval; MCS‐12, Mental Component Summary Scale Score; PCS‐12, Physical Component Summary Scale Score; SD, standard deviation

For maternal outcomes, there were no significant differences in physical component summary scale score (PCS‐12) and mental component summary scale score (MCS‐12) between women allocated to planned delivery and expectant management arms at 2 years (PCS‐12 mean difference 0.29, 95% CI −1.29 to 1.87; MCS‐12 mean difference 1.27, 95% CI −0.86 to 3.40) (Figure 3). Similar summary scores and subdomain scores were seen at 6 months and 2 years, indicating no evidence of a change of health status during follow‐up.

### 3.4 Sensitivity analyses (infant outcomes)

Sensitivity analyses including only infants assessed within a corrected age range of 23.5–27.5 months did not alter the findings (Tables S4 and S5).

### 3.5 Subgroup analyses (infant outcomes)

Pre‐specified analyses for the PARCA‐R composite score did not suggest important clinical differences by subgroups for both ITT and PP populations (Figure S1).

### 3.6 Women responding to follow‐up

The baseline characteristics of responders and non‐responders at the 2‐year assessment are described in Tables S6 and S7. Maternal responders at the 2‐year follow‐up were more likely to be white, have a low deprivation index score and were less likely to currently smoke at the time of initial antenatal visit, compared with those who did not respond. Short‐term infant outcomes between responders and non‐responders at the 2‐year follow‐up were similar with regards to neonatal unit admission, birth of a small‐for‐gestational age (<10th centile) infant and short‐term morbidity (Table S2).

## 4 DISCUSSION

### 4.1 Main findings

The mean standardised PARCA‐R scores at 2 years for infants of mothers with late preterm pre‐eclampsia randomised to planned early delivery or expectant management indicate that, on average, their neurodevelopment is within the normal range for both trial groups.^9^ This provides reassuring data on the long‐term outcomes of infants born late preterm, even when the additional complication of pre‐eclampsia is present. Subgroup analysis by gestational age at randomisation showed that mean standardised scores remained within the normal range, even at earlier gestations (34^+0^–34^+6^ weeks of gestation), where the severity of disease may also be worse. The confidence intervals for the adjusted mean difference of −2.4 points in the planned delivery arm compared with the expectant management arm were above the pre‐specified threshold to be able to definitively confirm the non‐inferiority of planned delivery. However, a mean difference of two points is unlikely to be clinically important at 2 years of age. No evidence of a difference was found in quality of maternal mental or physical health at 6 months and at 2 years between the two groups. Mean SF12‐v2 scores were consistent with those previously reported in similar populations.^15^, ^16^

### 4.2 Strengths and limitations

This is the largest trial to date evaluating planned early delivery in late preterm pre‐eclampsia and provides important information for clinicians and women faced with this clinical scenario. Long‐term follow‐up was identified as an important component of the research question and every possible strategy was employed to maximise the number of respondents. Similar trials attempting long‐term follow‐up of women and their infants report response rates varying from 14% to 61%,^19^, ^20^, ^21^ demonstrating the challenge associated with this objective, particularly when the population of interest is generally healthy and not under routine clinical follow‐up (in contrast to infants born very preterm). Thus, the inclusion of long‐term outcome data is a strength of this study and is likely to be of interest to women with pre‐eclampsia and their clinicians.

The trial was limited by a higher loss to follow‐up rate than expected, meaning that the extent and direction of bias in outcomes (between responders and non‐responders) is uncertain. This was compounded by PARCA‐R questionnaires being sent out at chronological rather than corrected age, meaning that imputation was needed to convert some raw scores into standardised scores. With a smaller sample size than expected for the long‐term primary outcome, and the consequently reduced precision of our estimates, our ability to draw firm conclusions is limited. A longer follow‐up period (e.g. up to 5 years) would have enabled us to provide further evidence on long‐term infant outcomes, using measures such as intelligence quotient (IQ), and to identify whether any of the differences observed between the two groups resulted in any clinical meaningful differences at school age, but this runs the risk of greater attrition and increased expense.

### 4.3 Interpretation

Infants born late preterm have been found to be at increased risk of neurodevelopmental delay and poor school performance in the long term,^22^, ^23^, ^24^, ^25^, ^26^ but this is typically compared with healthy infants born at term.^27^ Pre‐eclampsia is a disease state associated with fetal growth restriction,^28^ which itself is demonstrated to adversely impact childhood development.^29^, ^30^ In this scenario, it is possible that earlier delivery might improve long‐term neonatal outcomes, compared with expectant management which is associated with increased risk of growth restriction.^7^, ^20^, ^31^ In support of this, previous trials have shown that although infants of women with hypertensive disorders of pregnancy who underwent planned early delivery between 34^+0^ and 36^+6^ weeks of gestation had a small difference in neurodevelopmental outcomes at 2 years of age,^20^ these differences did not persist at the 5‐year follow‐up.^21^ At 5 years of age, other factors such as maternal education and birthweight appear to be more important predictors of long‐term infant development than near‐term gestational age at delivery.^21^, ^26^

This trial provides strong evidence that planned early delivery reduces immediate adverse maternal outcomes with no evidence of differences in self‐reported quality of maternal physical and mental health at 6 months and at 2 years between the intervention groups. However, the impact upon the infant remains unclear. Planned early delivery may increase the need for neonatal unit admission in the short term, primarily for an indication of prematurity (i.e. a routine admission without objective morbidity), but there is no evidence that it increases short‐term neonatal morbidity. At 2 years, the mean PARCA‐R scores for infants across both groups were within the normal range, which suggests no clinically important long‐term harm to the infant, but as the confidence intervals for the mean difference between the groups crosses the pre‐specified non‐inferiority margin, uncertainty remains. Pre‐eclampsia is an independent risk factor for adverse infant neurodevelopmental outcomes,^26^, ^32^, ^33^, ^34^ and the mean PARCA‐R scores in this trial were at the lower end of the normal range, consistent with previous studies. Infants in the planned early delivery group had lower PARCA‐R scores compared with those in the expectant management group, but the mean difference of −2.4 points is unlikely to be clinically meaningful or to influence longer‐term outcomes, such as school performance, particularly once other important predictors such as socio‐economic status are taken into account.^26^ In addition, the risks for an infant associated with late preterm birth must be balanced against those associated with continuing fetal growth restriction.

Future research must focus on how best to communicate these findings to women and translate them into clinical practice. The choice of clinically meaningful neonatal outcomes, particularly for infants born to mothers with pre‐eclampsia, remains a challenge and an area where further work and consensus building is needed.^18^ Furthermore, an intervention such as planned early delivery is likely to have a considerably different impact in different contexts where resources and disease burden are different. Most maternal and perinatal deaths associated with pre‐eclampsia occur in low‐ and middle‐income countries,^35^ which have markedly higher stillbirth rates than those reported in high‐income healthcare settings.^36^ A multicentre randomised controlled trial evaluating the effect of planned delivery on adverse maternal outcomes and perinatal morbidity and mortality is currently underway.^37^

## 5 CONCLUSION

Our results show that in women with late preterm pre‐eclampsia, the average neurodevelopmental assessment of infants at 2 years lies within the normal range, regardless of the timing of delivery. The small between‐group difference in PARCA‐R scores is unlikely to be clinically important, but because of the lower than anticipated follow‐up rate there was limited power to demonstrate that these scores did not differ. This follow‐up provides further information for clinicians about the balance of risks of benefits of planned early delivery between 34^+0^ and 36^+6^ weeks of gestation to facilitate shared decision making.

## CONFLICT OF INTEREST

NM reports personal fees from Shire and Novartis, outside of the submitted work. All other authors report no conflicts of interests. Completed disclosure of interests form available to view online as supporting information.

## AUTHOR CONTRIBUTIONS

LCC, RH, EJ, NM and AS were involved in the study conception and in securing funding for the study. LCC and AS were co‐chief investigators, responsible for all aspects of the study. LL supervised the study analyses, with input from LCC. MG performed the study analysis. AP made a substantial contribution to the running of the trial. RH did the health economic analysis. AB‐G, MG and LCC wrote the article. All authors reviewed, contributed to and approved the final version for publication.

## ETHICAL APPROVAL

The trial was approved by the South Central – Hampshire B Research Ethics Committee (no. 13/SC/0645).

## Supporting information


Supplementary material

Supplementary material

Supplementary material

Supplementary material

Supplementary material

Supplementary material

Supplementary material

Supplementary material

Supplementary material

Supplementary material

Supplementary material

Supplementary material

Supplementary material

## Data Availability

The data that support the findings of this study are available from the corresponding author, upon reasonable request.

## References

[1] Roberts CL , Ford JB , Algert CS , Antonsen S , Chalmers J , Cnattingius S , et al. Population‐based trends in pregnancy hypertension and pre‐eclampsia: an international comparative study. BMJ Open. 2011;1(1):e000101. 10.1136/bmjopen-2011-000101 PMC3191437 22021762

[2] Duley L . The global impact of pre‐eclampsia and eclampsia. Semin Perinatol. 2009;33(3):130–7. 19464502 10.1053/j.semperi.2009.02.010

[3] Chappell LC , Cluver CA , Kingdom J , Tong S . Pre‐eclampsia. Lancet. 2021;398:341–54. 34051884 10.1016/S0140-6736(20)32335-7

[4] National Institute for Health and Care Excellence . Hypertension in pregnancy: diagnosis and management. 2019 [cited 2022 Jan 26]. Available from: www.nice.org.uk/guidance/ng133 31498578

[5] Brown MA , Magee LA , Kenny LC , Karumanchi SA , McCarthy FP , Saito S , et al. The hypertensive disorders of pregnancy: ISSHP classification, diagnosis & management recommendations for international practice. Pregnancy Hypertens. 2018;13:291–310. 29803330 10.1016/j.preghy.2018.05.004

[6] Cluver C , Novikova N , Koopmans CM , West HM . Planned early delivery versus expectant management for hypertensive disorders from 34 weeks gestation to term. Cochrane Database Syst Rev. 2017;1:CD009273. 28106904 10.1002/14651858.CD009273.pub2 PMC6465052

[7] Chappell LC , Brocklehurst P , Green ME , Hunter R , Hardy P , Juszczak E , et al. Planned early delivery or expectant management for late preterm pre‐eclampsia (PHOENIX): a randomised controlled trial. Lancet. 2019;394(10204):1181–90. 31472930 10.1016/S0140-6736(19)31963-4 PMC6892281

[8] Chappell LC , Green M , Marlow N , Sandall J , Hunter R , Robson S , et al. Planned delivery or expectant management for late preterm pre‐eclampsia: study protocol for a randomised controlled trial (PHOENIX trial). Trials. 2019;20(1):85. 30691508 10.1186/s13063-018-3150-1 PMC6350286

[9] Johnson SBV , Linsell L , Brocklehurst P , Marlow N , Wolke D , Manktelow B . Parent Report of Children's Abilities – Revised (PARCA‐R). technical and interpretive manual. Leicester: University of Leicester; 2019. 10.1016/S2352-4642(19)30189-0 31402196

[10] National Institute for Health and Care Excellence . Developmental follow‐up of children and young people born preterm, 2017. 28837304

[11] Johnson S , Bountziouka V , Brocklehurst P , Linsell L , Marlow N , Wolke D , et al. Standardisation of the parent report of Children's Abilities‐Revised (PARCA‐R): a norm‐referenced assessment of cognitive and language development at age 2 years. Lancet Child Adolesc Health. 2019;3(10):705–12. 31402196 10.1016/S2352-4642(19)30189-0

[12] Ware J Jr , Kosinski M , Keller SD . A 12‐Item Short‐Form Health Survey: construction of scales and preliminary tests of reliability and validity. Med Care. 1996;34(3):220–33. 8628042 10.1097/00005650-199603000-00003

[13] Morrell CJ , Slade P , Warner R , Paley G , Dixon S , Walters SJ , et al. Clinical effectiveness of health visitor training in psychologically informed approaches for depression in postnatal women: pragmatic cluster randomised trial in primary care. BMJ. 2009;338:a3045. 19147636 10.1136/bmj.a3045 PMC2628298

[14] Emmanuel E , St John W , Sun J . Relationship between social support and quality of life in childbearing women during the perinatal period. J Obstet Gynecol Neonatal Nurs. 2012;41(6):E62–70. 10.1111/j.1552-6909.2012.01400.x 22861382

[15] Norhayati MN , Nik Hazlina NH , Aniza AA . Immediate and long‐term relationship between severe maternal morbidity and health‐related quality of life: a prospective double cohort comparison study. BMC Public Health. 2016;16(1):818. 27538506 10.1186/s12889-016-3524-9 PMC4990872

[16] Vinturache A , Stephenson N , McDonald S , Wu M , Bayrampour H , Tough S . Health‐related quality of life in pregnancy and postpartum among women with assisted conception in Canada. Fertil Steril. 2015;104(1):188–95.e1. 25956365 10.1016/j.fertnstert.2015.04.012

[17] von Dadelszen P , Payne B , Li J , Ansermino JM , Pipkin FB , Côté AM , et al. Prediction of adverse maternal outcomes in pre‐eclampsia: development and validation of the fullPIERS model. Lancet. 2011;377(9761):219–27. 21185591 10.1016/S0140-6736(10)61351-7

[18] Duffy J , Cairns AE , Richards‐Doran D , 't Hooft J , Gale C , Brown M , et al. A core outcome set for pre‐eclampsia research: an international consensus development study. BJOG. 2020;127:1516–26. 32416644 10.1111/1471-0528.16319

[19] Brocklehurst P , Field D , Greene K , Juszczak E , Keith R , Kenyon S , et al. Computerised interpretation of fetal heart rate during labour (INFANT): a randomised controlled trial. Lancet. 2017;389(10080):1719–29. 28341515 10.1016/S0140-6736(17)30568-8 PMC5413601

[20] Zwertbroek EF , Franssen MTM , Broekhuijsen K , Langenveld J , Bremer H , Ganzevoort W , et al. Neonatal developmental and behavioral outcomes of immediate delivery versus expectant monitoring in mild hypertensive disorders of pregnancy: 2‐year outcomes of the HYPITAT‐II trial. Am J Obstet Gynecol. 2019;221(2):154.e1–11. 30940558 10.1016/j.ajog.2019.03.024

[21] Zwertbroek EF , Zwertbroek J , Broekhuijsen K , Franssen MTM , Ganzevoort W , Langenveld J , et al. Neonatal developmental and behavioral outcomes of immediate delivery versus expectant monitoring in mild hypertensive disorders of pregnancy: 5‐year outcomes of the HYPITAT II trial. Eur J Obstet Gynecol Reprod Biol. 2020;244:172–9. 31810023 10.1016/j.ejogrb.2019.11.001

[22] Johnson S , Waheed G , Manktelow BN , Field DJ , Marlow N , Draper ES , et al. Differentiating the preterm phenotype: distinct profiles of cognitive and behavioral development following late and moderately preterm birth. J Pediatr. 2018;193:85–92.e1. 29254758 10.1016/j.jpeds.2017.10.002

[23] Petrini JRDT , McCormick MC , Massolo ML , Green NS , Escobar GJ . Increased risk of adverse neurological development for late preterm infants. J Pediatr. 2009;154:169–76. 19081113 10.1016/j.jpeds.2008.08.020

[24] Murray SR , Shenkin SD , McIntosh K , Lim J , Grove B , Pell JP , et al. Long term cognitive outcomes of early term (37‐38 weeks) and late preterm (34‐36 weeks) births: a systematic review. Wellcome Open Res. 2017;2:101. 29387801 10.12688/wellcomeopenres.12783.1 PMC5721566

[25] Moster DLR , Markestad T . Long term medical and social consequences of preterm birth. N Engl J Med. 2008;359(3):262–73. 18635431 10.1056/NEJMoa0706475

[26] Johnson S , Evans TA , Draper ES , Field DJ , Manktelow BN , Marlow N , et al. Neurodevelopmental outcomes following late and moderate prematurity: a population‐based cohort study. Arch Dis Child Fetal Neonatal Ed. 2015;100(4):F301–8. 25834170 10.1136/archdischild-2014-307684 PMC4484499

[27] Teune MJ , Bakhuizen S , Gyamfi Bannerman C , Opmeer BC , van Kaam AH , van Wassenaer AG , et al. A systematic review of severe morbidity in infants born late preterm. Am J Obstet Gynecol. 2011;205(4):374.e1–9. 21864824 10.1016/j.ajog.2011.07.015

[28] Burton GJ , Redman CW , Roberts JM , Moffett A . Pre‐eclampsia: pathophysiology and clinical implications. BMJ. 2019;366:l2381. 31307997 10.1136/bmj.l2381

[29] Figueras F , Eixarch E , Meler E , Iraola A , Figueras J , Puerto B , et al. Small‐for‐gestational‐age fetuses with normal umbilical artery doppler have suboptimal perinatal and neurodevelopmental outcome. Eur J Obstet Gynecol Reprod Biol. 2008;136(1):34–8. 17434250 10.1016/j.ejogrb.2007.02.016

[30] van Wyk L , Boers KE , van der Post JA , van Pampus MG , van Wassenaer AG , van Baar AL , et al. Effects on (neuro)developmental and behavioral outcome at 2 years of age of induced labor compared with expectant management in intrauterine growth‐restricted infants: long‐term outcomes of the DIGITAT trial. Am J Obstet Gynecol. 2012;206(5):406.e1–7. 22444791 10.1016/j.ajog.2012.02.003

[31] Boers KE , Vijgen SM , Bijlenga D , van der Post JAM , Bekedam DJ , Kwee A , et al. Induction versus expectant monitoring for intrauterine growth restriction at term: randomised equivalence trial (DIGITAT). BMJ. 2010;341:c7087. 21177352 10.1136/bmj.c7087 PMC3005565

[32] Ananth CV , Friedman AM . Ischemic placental disease and risks of perinatal mortality and morbidity and neurodevelopmental outcomes. Semin Perinatol. 2014;38(3):151–8. 24836827 10.1053/j.semperi.2014.03.007

[33] Habli M , Levine RJ , Qian C , Sibai B . Neonatal outcomes in pregnancies with preeclampsia or gestational hypertension and in normotensive pregnancies that delivered at 35, 36, or 37 weeks of gestation. Am J Obstet Gynecol. 2007;197(4):406.e1–7. 17904980 10.1016/j.ajog.2007.06.059

[34] Warshafsky C , Pudwell J , Walker M , Wen S‐W , Smith GN . Prospective assessment of neurodevelopment in children following a pregnancy complicated by severe pre‐eclampsia. BMJ Open. 2016;6(7):e010884. 10.1136/bmjopen-2015-010884 PMC4947739 27388354

[35] Say L , Chou D , Gemmill A , Tunçalp Ö , Moller AB , Daniels J , et al. Global causes of maternal death: a WHO systematic analysis. Lancet Glob Health. 2006;2:e323–33. 10.1016/S2214-109X(14)70227-X 25103301

[36] Nathan HL , Seed PT , Hezelgrave NL , de Greeff A , Lawley E , Conti‐Ramsden F , et al. Maternal and perinatal adverse outcomes in women with pre‐eclampsia cared for at facility‐level in South Africa: a prospective cohort study. J Glob Health. 2018;8(2):020401. 30140431 10.7189/jogh.08-020401 PMC6076583

[37] Beardmore‐Gray A , Vousden N , Charantimath U , Katageri G , Bellad M , Kapembwa K , et al. Planned early delivery versus expectant management to reduce adverse pregnancy outcomes in pre‐eclampsia in a low‐ and middle‐income setting: study protocol for a randomised controlled trial (CRADLE‐4 trial). Trials. 2020;21(1):960. 33228794 10.1186/s13063-020-04888-w PMC7684962

