# Supplementary material for: Two‐year follow‐up of infant and maternal outcomes after planned early delivery or expectant management for late preterm pre‐eclampsia (PHOENIX): A randomised controlled trial
Source: BJOG. 2022 May 12;129(10):1654–63. doi: 10.1111/1471-0528.17167 (PMC9545311; doi:10.1111/1471-0528.17167)
Supplement: Supplementary file 1 — Supplementary material [file BJO-129-1654-s009.docx]

### Table S1: Centre at randomisation of responders at two-year assessment*

| **Name** | **Planned delivery (n=276)** | **Expectant management (n=251)** |
| --- | --- | --- |
| St Thomas' Hospital, London | 19 (6.9) | 22 (8.8) |
| Darent Valley Hospital | 17 (6.2) | 15 (6.0) |
| St Mary's Hospital, Manchester | 17 (6.2) | 10 (4.0) |
| Bradford Royal Infirmary | 8 (2.9) | 4 (1.6) |
| West Middlesex University | 17 (6.2) | 15 (6.0) |
| Nottingham City Hospital | 9 (3.3) | 9 (3.6) |
| Leeds Teaching Hospitals - St James' | 14 (5.1) | 6 (2.4) |
| Liverpool Women's | 11 (4.0) | 13 (5.2) |
| Queens Medical Centre | 6 (2.2) | 5 (2.0) |
| Royal Victoria Infirmary | 7 (2.5) | 10 (4.0) |
| James Cook University Hospital | 9 (3.3) | 15 (6.0) |
| Sunderland Royal Hospital | 11 (4.0) | 16 (6.4) |
| University College Hospital | 6 (2.2) | 10 (4.0) |
| Birmingham Women's Hospital | 6 (2.2) | 3 (1.2) |
| St George's Hospital | 5 (1.8) | 0 (0.0) |
| Royal Stoke University Hospital | 6 (2.2) | 4 (1.6) |
| Western Sussex Hospitals | 5 (1.8) | 9 (3.6) |
| Whittington Hospital | 3 (1.1) | 2 (0.8) |
| ABM University Hospitals, Wales | 16 (5.8) | 11 (4.4) |
| Birmingham City Hospital | 6 (2.2) | 2 (0.8) |
| Birmingham Heartlands Hospital | 4 (1.4) | 0 (0.0) |
| Warrington and Halton Hospitals | 2 (0.7) | 2 (0.8) |
| Chesterfield Royal Hospital | 2 (0.7) | 3 (1.2) |
| Royal United Hospital, Bath | 3 (1.1) | 3 (1.2) |
| Kingston Hospital NHS Trust | 11 (4.0) | 9 (3.6) |
| Leighton Hospital | 4 (1.4) | 6 (2.4) |
| Leicester Royal infirmary | 6 (2.2) | 6 (2.4) |
| Shrewsbury and Telford Hospital | 0 (0.0) | 3 (1.2) |
| Royal Preston Hospital | 1 (0.4) | 3 (1.2) |
| Northampton General | 5 (1.8) | 3 (1.2) |
| Gloucestershire Royal Hospital | 0 (0.0) | 1 (0.4) |
| St Michael's Hospital, Bristol | 2 (0.7) | 3 (1.2) |
| Royal London Hospital | 4 (1.4) | 1 (0.4) |
| Whipps Cross Hospital | 4 (1.4) | 4 (1.6) |
| New Cross Hospital,Wolverhampton | 3 (1.1) | 2 (0.8) |
| Cambridge University Hospitals | 2 (0.7) | 0 (0.0) |
| Chelsea and Westminster Hospital | 0 (0.0) | 3 (1.2) |
| Royal Bolton Hospital | 2 (0.7) | 1 (0.4) |
| St Helier Hospital | 2 (0.7) | 1 (0.4) |
| University Hospital, Lewisham | 0 (0.0) | 2 (0.8) |
| Epsom Hospital | 1 (0.4) | 0 (0.0) |
| Queen Elizabeth Hospital, Greenwich | 4 (1.4) | 0 (0.0) |
| Queen's Hospital, Romford | 2 (0.7) | 2 (0.8) |
| Croydon University Hospital | 11 (4.0) | 12 (4.8) |
| Broomfield Hospital, Chelmsford | 3 (1.1) | 0 (0.0) |

### Table S2: Short-term infant outcomes prior to hospital discharge home of responders at two-year assessment and non-responders

|  | **Planned delivery** | **Expectant mangement** |  | **Planned delivery** | **Expectant mangement** |
| --- | --- | --- | --- | --- | --- |
|  | **Responders (n=290)** | **Responders (n=256)** |  | **Non-responders (n=181)** | **Non-responders (n=219)** |
| Gestational age at delivery (days), median (IQR) | 253 (247, 257) | 258 (251, 260) |  | 251 (245, 257) | 257 (252, 260) |
| 34 weeks | 50 (17.2) | 30 (11.7) |  | 39 (21.8) | 17 (7.8) |
| 35 weeks | 88 (30.3) | 42 (16.4) |  | 51 (28.5) | 34 (15.5) |
| 36 weeks | 96 (33.1) | 70 (27.3) |  | 63 (35.2) | 68 (31.1) |
| ≥37 weeks | 56 (19.3) | 114 (44.5) |  | 26 (14.5) | 100 (45.7) |
| Missing | 0 | 0 |  | 2 | 0 |
| Mode of birth |  |  |  |  |  |
| Spontaneous vaginal | 101 (34.8) | 71 (27.7) |  | 68 (38.0) | 68 (31.1) |
| Assisted vaginal | 28 (9.7) | 24 (9.4) |  | 12 (6.7) | 23 (10.5) |
| Caesarean section | 161 (55.5) | 161 (62.9) |  | 99 (55.3) | 128 (58.4) |
| Missing | 0 | 0 |  | 2 | 0 |
| Birth weight (g), median (IQR) | 2430  (2112 to 2775) | 2438  (2150 to 2820) |  | 2390  (2006 to 2753) | 2510  (2160 to 3078) |
| Missing | 0 | 0 |  | 2 | 0 |
| Birthweight centile, median (IQR)* | 37 (17 to 60) | 30 (12 to 56) |  | 34 (15 to 65) | 32 (15 to 67) |
| <10th centile, n(%) | 41 (14.1) | 55 (21.5) |  | 33 (18.4) | 40 (18.3) |
| <3rd centile, n(%) | 8 (2.8) | 13 (5.1) |  | 12 (6.7) | 14 (6.4) |
| Missing | 0 | 0 |  | 2 | 0 |
| Apgar score at 5 minutes after birth, median (IQR) | 10 (9 to 10) | 10 (9 to 10) |  | 9 (9 to 10) | 10 (9 to 10) |
| Missing | 0 | 0 |  | 2 | 0 |
| Umbilical arterial pH collected | 175 (60.3) | 147 (57.4) |  | 106 (58.6) | 119 (54.3) |
| Median (IQR) | 7 (7 to 7) | 7 (7 to 7) |  | 7 (7 to 7) | 7 (7 to 7) |
| Missing | 0 | 2 |  | 2 | 1 |
| Admission to neonatal unit | 117 (40.3) | 91 (35.5) |  | 79 (44.1) | 68 (31.1) |
| Missing | 0 | 0 |  | 2 | 0 |
| Principal recorded indication for neonatal unit admission, n (%) |  |  |  |  |  |
| Prematurity | 50 (42.7) | 25 (27.5) |  | 33 (41.8) | 15 (22.1) |
| Respiratory disease | 31 (26.5) | 18 (19.8) |  | 16 (20.3) | 23 (33.8) |
| Cardiovascular disease | 0 (0.0) | 1 (1.1) |  | 0 (0.0) | 0 (0.0) |
| Failed oximetry testing | 0 (0.0) | 1 (1.1) |  | 0 (0.0) | 0 (0.0) |
| Jaundice | 6 (5.1) | 7 (7.7) |  | 6 (7.6) | 4 (5.9) |
| Hypoglycaemia | 8 (6.8) | 20 (22.0) |  | 13 (16.5) | 11 (16.2) |
| Convulsions suspected/confirmed | 0 (0.0) | 0 (0.0) |  | 1 (1.3) | 0 (0.0) |
| Poor condition at birth | 1 (0.9) | 1 (1.1) |  | 1 (1.3) | 2 (2.9) |
| Infection suspected/confirmed | 9 (7.7) | 7 (7.7) |  | 0 (0.0) | 5 (7.4) |
| IUGR/ SGA | 3 (2.6) | 5 (5.5) |  | 5 (6.3) | 5 (7.4) |
| Poor feeding or weight loss | 4 (3.4) | 2 (2.2) |  | 0 (0.0) | 0 (0.0) |
| Congenital anomaly suspected/confirmed | 2 (1.7) | 0 (0.0) |  | 0 (0.0) | 0 (0.0) |
| Maternal admission/emergency | 1 (0.9) | 1 (1.1) |  | 0 (0.0) | 1 (1.5) |
| Monitoring | 2 (1.7) | 3 (3.3) |  | 2 (2.5) | 2 (2.9) |
| Continuing care | 0 (0.0) | 0 (0.0) |  | 2 (2.5) | 0 (0.0) |
| Need for respiratory support, n (%) | 28 (9.7) | 21 (8.2) |  | 17 (9.5) | 27 (12.3) |
| Missing | 0 | 0 |  | 2 | 0 |
| Need for supplementary oxygen prior to discharge, n (%) | 40 (13.8) | 22 (8.6) |  | 20 (11.2) | 27 (12.3) |
| Missing | 0 | 0 |  | 2 | 0 |
| Number of days supplemental oxygen required, median (IQR) | 1 (1 to 2) | 2 (1 to 3) |  | 2 (1 to 3) | 1 (1 to 4) |
| Range (min to max) | (0 to 7) | (1 to 11) |  | (1 to 12) | (0 to 31) |
| Missing | 250 | 234 |  | 161 | 192 |
| Total time in neonatal unit (days), median (IQR) | 5 (3 to 8) | 4 (3 to 8) |  | 5 (3 to 8) | 4 (2 to 7) |
| Number admitted for at least 1 day, n (%) | 109 (37.6) | 87 (34.0) |  | 72 (39.8) | 66 (30.1) |
| Category of care during neonatal unit stay (separation of baby from mother) |  |  |  |  |  |
| Intensive care, n (%) | 12 (4.1) | 9 (3.6) |  | 15 (8.4) | 10 (4.6) |
| Days, median (IQR) | 1 (1 to 2) | 2 (1 to 2) |  | 2 (1 to 3) | 4 (3 to 5) |
| High dependency care, n (%) | 33 (11.4) | 24 (9.5) |  | 18 (10.1) | 9 (4.1) |
| Days, median (IQR) | 1 (1 to 3) | 2 (1 to 5) |  | 2 (1 to 2) | 2 (1 to 4) |
| Special care (carer not present), n (%) | 101 (34.8) | 80 (31.7) |  | 67 (37.4) | 63 (29.0) |
| Days, median (IQR) | 5 (2 to 9) | 7 (2 to 11) |  | 6 (3 to 11) | 5 (2 to 10) |
| Category of care during other postnatal stay (baby alongside mother) |  |  |  |  |  |
| Transitional care (special care with carer present), n (%) | 24 (8.3) | 8 (3.2) |  | 16 (8.9) | 8 (3.7) |
| Days, median (IQR) | 6 (2 to 9) | 5 (4 to 6) |  | 4 (2 to 6) | 5 (4 to 6) |
| Postnatal care, n(%) | 216 (74.5) | 204 (81.0) |  | 134 (74.9) | 180 (82.9) |
| Days, median (IQR) | 3 (2 to 5) | 3 (2 to 4) |  | 3 (2 to 4) | 3 (2 to 5) |

IUGR: intrauterine growth restriction. SGA: Small-for-gestational age. *Birthweight centile calculated using the Stata add-in function zanthro using the British 1990 Growth Reference (reanalysed 2009).

### Table S3: Short-term maternal outcomes prior to hospital discharge home of responders at two-year assessment and non-responders

|  | **Planned delivery** | **Expectant mangement** |  | **Planned delivery** | **Expectant mangement** |
| --- | --- | --- | --- | --- | --- |
|  | **Responders (n=276)** | **Responders (n=251)** |  | **Non-responders (n=172)** | **Non-responders (n=200)** |
| Maternal co-primary outcome (maternal morbidity composite outcome or systolic blood pressure ≥160 mmHg post randomisation, n (%) | 180 (65.2) | 188 (75.5) |  | 109 (63.7) | 150 (75.0) |
| Missing | 0 | 2 |  | 1 | 0 |
| Maternal morbidity composite outcome, n (%) | 42 (15.2) | 47 (18.9) |  | 26 (15.2) | 43 (21.5) |
| Missing | 0 | 2 |  | 1 | 0 |
| Systolic blood pressure ≥160 mmHg post randomisation, n (%) | 165 (59.8) | 178 (71.8) |  | 102 (59.6) | 135 (67.5) |
| Missing | 0 | 3 |  | 1 | 0 |
| Progression to severe pre-eclampsia, n (%) | 180 (65.2) | 185 (74.3) |  | 107 (62.6) | 149 (74.5) |
| Missing | 0 | 2 |  | 1 | 0 |
| Placental abruption, n (%) | 3 (1.1) | 3 (1.2) |  | 1 (0.6) | 1 (0.5) |
| Missing | 0 | 2 |  | 1 | 0 |
| Antihypertensive medication before delivery, n (%) | 239 (86.6) | 225 (89.6) |  | 142 (83.0) | 180 (90.0) |
| Missing | 0 | 0 |  | 1 | 0 |
| Onset of labour, n (%) |  |  |  |  |  |
| Spontaneous | 1 (0.4) | 9 (3.6) |  | 1 (0.6) | 10 (5.0) |
| Induced | 190 (68.8) | 157 (62.8) |  | 114 (66.7) | 118 (59.0) |
| Pre-labour caesarean section | 84 (30.4) | 83 (33.2) |  | 56 (32.7) | 69 (34.5) |
| PROM and augmentation | 1 (0.4) | 1 (0.4) |  | 0 (0.0) | 3 (1.5) |
| Missing | 0 | 1 |  | 1 | 0 |
| Indication for delivery (non-exclusive) |  |  |  |  |  |
| Spontaneous labour <37 weeks gestation | 1 (0.4) | 9 (3.6) |  | 1 (0.6) | 10 (5.0) |
| Trial allocation to planned delivery arm | 275 (99.6) | 0 (0.0) |  | 170 (99.4) | 0 (0.0) |
| Reaching 37 weeks' gestation | 5 (1.8) | 115 (46.2) |  | 3 (1.8) | 73 (36.5) |
| Uncontrolled maternal hypertension | 16 (5.8) | 66 (26.5) |  | 10 (5.8) | 45 (22.5) |
| Maternal haematological abnormality | 1 (0.4) | 8 (3.2) |  | 2 (1.2) | 15 (7.5) |
| Maternal biochemical abnormality | 10 (3.6) | 28 (11.2) |  | 9 (5.3) | 29 (14.5) |
| Fetal compromise on ultrasound scan | 8 (2.9) | 20 (8.0) |  | 8 (4.7) | 30 (15.0) |
| Fetal compromise on cardiotocography | 18 (6.5) | 37 (14.9) |  | 15 (8.8) | 27 (13.5) |
| Severe maternal symptoms | 5 (1.8) | 27 (10.8) |  | 4 (2.3) | 21 (10.5) |
| Other (with none of the above) | 0 (0.0) | 2 (0.8) |  | 0 (0.0) | 0 (0.0) |
| Missing | 0 | 2 |  | 1 | 0 |
| Maternal complications between randomisation and discharge |  |  |  |  |  |
| Confirmed thromboembolic disease, n (%) | 0 (0.0) | 0 (0.0) |  | 0 (0.0) | 0 (0.0) |
| Confirmed sepsis (positive blood or urine cultures), n (%) | 0 (0.0) | 4 (1.6) |  | 2 (1.2) | 2 (1.0) |

PROM: Pre-labour rupture of membranes

### Table S4: Ages and response times at two years for infants

|  | **Planned delivery  (n=290)** | **Expectant management  (n=256)** |
| --- | --- | --- |
| Chronological age at two year assessment (days) |  |  |
| Mean (SD) | 734 (41.5) | 734 (41.6) |
| Median (IQR) | 723 (710 to 745) | 721 (706 to 746) |
| Age corrected for prematurity at two year assessment (days) |  |  |
| Mean (SD) | 706 (42.2) | 709 (42.8) |
| Median (IQR) | 696 (680 to 716) | 697 (684 to 723) |
| Age corrected for prematurity time window for completing two year assessment |  |  |
| 17 months 15 days - 18 months 14 days | 1 (0.3) | 0 (0.0) |
| 20 months 15 days - 21 months 14 days | 1 (0.3) | 1 (0.4) |
| 21 months 15 days - 22 months 14 days | 90 (31.0) | 72 (28.1) |
| 22 months 15 days - 23 months 14 days | 125 (43.1) | 101 (39.5) |
| **23 months 15 days - 24 months 14 days** | **33 (11.4)** | **44 (17.2)** |
| **24 months 15 days - 25 months 14 days** | **17 (5.9)** | **18 (7.0)** |
| **25 months 15 days - 26 months 14 days** | **11 (3.8)** | **10 (3.9)** |
| **26 months 15 days - 27 months 14 days** | **7 (2.4)** | **7 (2.7)** |
| 27 months 15 days - 28 months 14 days | 3 (1.0) | 0 (0.0) |
| 28 months 15 days - 29 months 14 days | 1 (0.3) | 1 (0.4) |
| 29 months 15 days - 30 months 14 days | 1 (0.3) | 2 (0.8) |

Standardised PARCA-R scores are calculated within the 23.5 to 27.5 months’ time window for an infant’s age corrected for prematurity at two years.

### Table S5: Sensitivity analysis of primary infant long-term outcome non-inferiority comparison excluding infants assessed outside 23.5 to 27.5 months corrected age: Standardised Parent Report of Children’s Abilities Revised (PARCA-R) at two years follow-up

|  | **Planned delivery** | | **Expectant management** | | **Adjusted mean difference*  (95% CI)** |
| --- | --- | --- | --- | --- | --- |
|  | **N** | **Adjusted mean (SD)** | **N** | **Adjusted mean (SD)** |  |
| **Intention-to-treat** | (N=68) |  | (N=79) |  |  |
| Composite Score | 66 | 90.9 (16.4) | 72 | 95.2 (16.4) | -4.25 (-9.76,1.27) |
| Non-verbal subscale score | 67 | 95.9 (19.1) | 77 | 103.3 (19.1) | -7.39 (-13.69,-1.10) |
| Language subscale score | 67 | 90.2 (17.1) | 74 | 93.9 (17.1) | -3.63 (-9.32,2.05) |
|  |  |  |  |  |  |
| **Per-protocol** | (N=50) |  | (N=78) |  |  |
| Composite Score | 48 | 90.7 (16.0) | 72 | 94.8 (16.0) | -4.05 (-9.92,1.83) |
| Non-verbal subscale score | 49 | 96.8 (19.6) | 76 | 103.2 (19.6) | -6.36 (-13.24,0.52) |
| Language subscale score | 49 | 89.9 (16.8) | 74 | 93.5 (16.7) | -3.57 (-9.66,2.52) |

SD: standard deviation; CI: confidence interval.

### Figure S1: Subgroup analyses of the primary infant long-term outcome non-inferiority comparison: Imputed Standardised Parent Report of Children’s Abilities Revised (PARCA-R) at two years

A: Intention-to-treat analysis


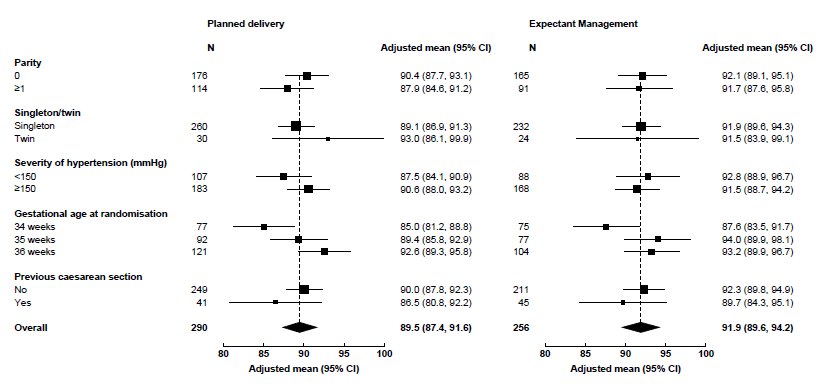


B: Per-protocol analysis


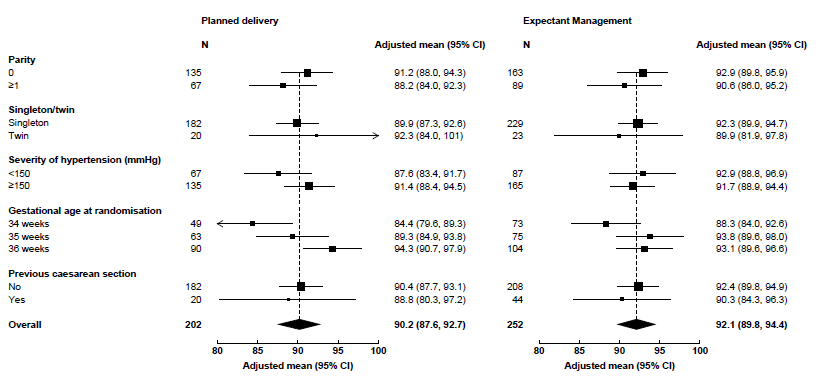


The dotted line indicates the overall adjusted mean by group.

### Table S6: Maternal baseline characteristics of responders at two-year assessment and non-responders

|  | **Planned delivery** | **Expectant mangement** |  | **Planned delivery** | **Expectant mangement** |
| --- | --- | --- | --- | --- | --- |
|  | **Responders (n=276)** | **Responders (n=251)** |  | **Non-responders (n=172)** | **Non-responders (n=200)** |
| Age at randomisation (years), mean (SD) | 31.1 (5.74) | 31.4 (6.10) |  | 29.7 (7.25) | 30.1 (6.50) |
| Ethnicity, n (%) |  |  |  |  |  |
| White | 200 (44.6) | 189 (41.9) |  | 113 (25.2) | 122 (27.1) |
| Mixed | 7 (1.6) | 11 (2.4) |  | 3 (0.7) | 12 (2.7) |
| Asian | 42 (9.4) | 22 (4.9) |  | 18 (4.0) | 28 (6.2) |
| Chinese | 0 (0.0) | 1 (0.2) |  | 0 (0.0) | 0 (0.0) |
| Black | 23 (5.1) | 21 (4.7) |  | 35 (7.8) | 31 (6.9) |
| Other | 3 (0.7) | 6 (1.3) |  | 2 (0.4) | 7 (1.6) |
| Unknown | 1 (0.2) | 1 (0.2) |  | 1 (0.2) | 0 (0.0) |
| Deprivation Index quintile, n (%)* |  |  |  |  |  |
| England |  |  |  |  |  |
| 1 (Least deprived) | 35 (13.6) | 20 (8.3) |  | 6 (3.8) | 5 (2.7) |
| 2 | 37 (14.3) | 33 (13.6) |  | 16 (10.0) | 20 (10.8) |
| 3 | 45 (17.4) | 48 (19.8) |  | 19 (11.9) | 24 (12.9) |
| 4 | 64 (24.8) | 63 (26.0) |  | 42 (26.3) | 55 (29.6) |
| 5 (Most deprived) | 79 (30.6) | 75 (31.0) |  | 82 (51.3) | 85 (45.7) |
| Missing | 0 | 1 |  | 0 | 0 |
| Wales unavailable | 16 | 11 |  | 7 | 11 |
| Parity (previous pregnancies ≥24 weeks' gestation)*, n (%) |  |  |  |  |  |
| 0 | 166 (37.1) | 159 (35.3) |  | 88 (19.6) | 101 (22.4) |
| 1 | 66 (14.7) | 52 (11.5) |  | 38 (8.5) | 51 (11.3) |
| 2 | 30 (6.7) | 28 (6.2) |  | 19 (4.2) | 24 (5.3) |
| >2 | 14 (3.1) | 12 (2.7) |  | 27 (6.0) | 24 (5.3) |
| Previous caesarean section**, n (%) | 40 (14.5) | 43 (17.1) |  | 38 (22.1) | 35 (17.5) |
| History of pre-eclampsia, n (%) | 50 (18.1) | 47 (18.7) |  | 36 (20.9) | 45 (22.5) |
| Body mass index at booking (kg/m^2^), mean (SD) | 30 (7.59) | 29.2 (6.70) |  | 29.5 (6.84) | 30.5 (7.79) |
| Smoking status at booking, n (%) |  |  |  |  |  |
| Never smoked | 214 (77.5) | 179 (71.3) |  | 113 (65.7) | 130 (65.0) |
| Quit before booking | 42 (15.2) | 52 (20.7) |  | 22 (12.8) | 34 (17.0) |
| Smoking at booking | 16 (5.8) | 16 (6.4) |  | 37 (21.5) | 34 (17.0) |
| Unknown | 4 (1.4) | 4 (1.6) |  | 0 (0.0) | 2 (1.0) |
| Blood pressure at booking (mmHg) |  |  |  |  |  |
| Systolic BP at booking (mmHg), mean (SD) | 119.0 (13.6) | 119.5 (13.2) |  | 118.3 (15.6) | 119.7 (14.3) |
| Diastolic BP at booking (mmHg), mean (SD) | 72.8 (10.0) | 73.3 (10.2) |  | 72.6 (10.6) | 73.6 (10.6) |
| Pre-existing chronic hypertension, n (%) | 29 (10.5) | 33 (13.1) |  | 22 (12.8) | 20 (10.0) |
| Pre-existing chronic renal disease, n (%) | 3 (1.1) | 2 (0.8) |  | 3 (1.7) | 2 (1.0) |
| Pre-pregnancy diabetes, n (%) | 15 (5.4) | 14 (5.6) |  | 10 (5.8) | 14 (7.0) |
| Gestational diabetes, n (%) | 36 (13.0) | 21 (8.4) |  | 26 (15.1) | 32 (16.0) |
| Aspirin prescribed during pregnancy, n (%) | 114 (41.3) | 101 (40.2) |  | 56 (32.6) | 88 (44.0) |
| LMWH prescribed during pregnancy, n (%) | 69 (25.0) | 66 (26.3) |  | 56 (32.6) | 51 (25.5) |

BP: blood pressure. LMWH: Low molecular weight heparin. *Deprivation quintiles calculated for participants in England only (not available for participants in Wales). **Minimisation factors used to ensure balance at randomisation.

### Table S7: Maternal characteristics at randomisation of responders at two-year assessment and non-responders

|  | **Planned delivery** | **Expectant mangement** |  | **Planned delivery** | **Expectant mangement** |
| --- | --- | --- | --- | --- | --- |
|  | **Responders (n=276)** | **Responders (n=251)** |  | **Non-responders (n=172)** | **Non-responders (n=200)** |
| Gestational age at randomisation* (weeks), median (IQR) | 36 (35 to 36) | 36 (35 to 36) |  | 35 (35 to 36) | 36 (35 to 36) |
| 34^+0^ to 34^+6^ | 73 (26.4) | 72 (28.7) |  | 58 (33.7) | 63 (31.5) |
| 35^+0^ to 35^+6^ | 85 (30.8) | 73 (29.1) |  | 52 (30.2) | 59 (29.5) |
| 36^+0^ to 36^+6^ | 118 (42.8) | 106 (42.2) |  | 62 (36.0) | 78 (39.0) |
| Number of live fetuses at study entry* |  |  |  |  |  |
| 1 | 261 (94.6) | 238 (94.8) |  | 164 (95.3) | 189 (94.5) |
| 2 | 15 (5.4) | 13 (5.2) |  | 8 (4.7) | 11 (5.5) |
| Highest systolic BP in previous 48hrs (mmHg), mean (SD) | 155 (14.8) | 155.6 (16.1) |  | 153.6 (13.9) | 154.6 (14.5) |
| Highest systolic BP in previous 48hrs (mmHg)**, n (%) |  |  |  |  |  |
| ≤149 | 100 (36.2) | 88 (35.1) |  | 63 (36.6) | 75 (37.5) |
| 150-159 | 69 (25.0) | 65 (25.9) |  | 52 (30.2) | 58 (29.0) |
| ≥160 | 107 (38.8) | 98 (39.0) |  | 57 (33.1) | 67 (33.5) |
| Highest diastolic BP in previous 48hrs (mmHg), mean (SD) | 95.8 (9.5) | 95.8 (11.3) |  | 95.6 (9.7) | 95.9 (8.4) |
| Urinary protein-creatinine ratio (after 20 weeks) ≥30 (mg/mol), n (%) | 253 (91.7) | 228 (90.8) |  | 152 (88.4) | 179 (89.5) |
| Most recent urinary protein-creatinine ratio (mg/mmol), median (IQR) | 88 (43 to 185) | 87 (43 to 197) |  | 78 (42 to 188) | 70 (40 to 152) |
| Missing | 0 | 0 |  | 0 | 1 |
| Fetal growth restriction ultrasound in previous two weeks, n (%) | 222 (80.4) | 212 (84.5) |  | 144 (83.7) | 163 (81.5) |
| Suspected fetal growth restriction | 44 (19.8) | 49 (23.1) |  | 35 (24.3) | 36 (22.1) |
| Bishop score at study entry |  |  |  |  |  |
| <2 | 1 (0.4) | 0 (0.0) |  | 1 (0.6) | 2 (1.0) |
| 2 to 6 | 4 (1.4) | 2 (0.8) |  | 3 (1.7) | 2 (1.0) |
| ≥6 | 0 (0.0) | 0 (0.0) |  | 0 (0.0) | 0 (0.0) |
| Cervix not assessed | 271 (98.2) | 249 (99.2) |  | 168 (97.7) | 196 (98.0) |
| In-patient at time of trial entry | 217 (78.6) | 210 (83.7) |  | 145 (84.3) | 161 (80.5) |

*Minimisation factors used to ensure balance at randomisation.

### Table S8: GRIPP2-SF Checklist

| Section and topic | Item | Reported on page No |
| --- | --- | --- |
| Aim | The aim of Public and Patient Involvement in all aspects of the study was to ensure that the voices of pregnant women (and their wider families) were woven throughout the research, such that the results would be of direct benefit to them. | 10 |
| Methods | We worked with Public and Patient Involvement representatives from grant preparation through to dissemination. As the study arose from a commissioned call, we were aware that pregnant women had been involved through the NICE guideline committee and the NIHR HTA prioritisation work, but we additionally worked with representatives (including those with lived experience) from Action on Pre-eclampsia (the patient support group) and Tommy’s Charity (a national baby charity). This involvement extended across considerations around research design, development and iteration of participant information resources, research management and troubleshooting (as members of the Co-Investigator Group and Trial Steering Committee), interpretation of the data, and writing and dissemination of the findings. | 10 |
| Study results | Examples of how PPI shaped the research included consideration of how to promote recruitment when it was slower than anticipated. A number of the central research team noted that women were often enthusiastic about participation, perceiving the clinical need for this uncertainty to be addressed, but that healthcare professionals could act as gatekeepers to enrolment. We worked with site teams to support them offering the trial to a greater proportion of eligible women, reinforcing that we had made the inclusion criteria as wide as possible for a pragmatic approach. We disseminated the information that around 55% of women who were approached agreed to take part, and with positive quotes from women (about participation) included in newsletters, with the women’s consent. This enabled a shift towards an inclusive approach to enrolment. | 10 |
| Discussion and conclusions | Pregnancy studies have had a long history of active PPI input, but for a timing of delivery trial this is particularly crucial as the trade-off between maternal and infant benefits and risks is central to the research question. PPI input has been pivotal around appropriate representation of this balance, accurate depiction of the existing equipoise, and interpretation of the findings when typically benefits may not always go in the same direction for the woman and the baby. PPI has only ever been a positive and essential guiding influence. | 10 |
| Reflections/critical perspective | The active involvement of Action on Pre-eclampsia, the patient support group, has been vital at all stages. This has enabled contribution through the Chief Executive Officer, Marcus Green, who combines indirect lived experience (as a partner of a woman with pre-eclampsia) with a powerful conduit to many other voices for whom he constantly advocates. The study has also had involvement of others with lived experience, but we noted that sometimes women transition through various phases of their lives and may choose to be involved for varying durations (not always for the entire length of the study). This has led Action on Pre-eclampsia to set up a research involvement panel, so that those with lived experience can contribute in the way that best suits them. | 10 |
